# Small Molecule Targeting of Chaperone {IP Protein} Prevents and Reverses TDP‐43 Aggregation In‐vitro and In‐vivo

**DOI:** 10.1002/alz70859_099984

**Published:** 2025-12-25

**Authors:** Marc Shenouda, Janice Robertson

**Affiliations:** ^1^ University of Toronto, Toronto, ON Canada; ^2^ Tanz Centre for Research in Neurodegenerative Diseases, Toronto, ON Canada

## Abstract

**Background:**

Nuclear depletion and neuronal cytoplasmic aggregation of the transactive response DNA‐binding protein 43 (TDP‐43) is observed in up to 95% amyotrophic lateral sclerosis (ALS) cases, 70% of Alzheimer’s Disease (AD) cases, and 50% of frontotemporal dementia (FTD) cases. The cytoplasmic aggregation of TDP‐43 has been correlated with inducing neuronal loss. As such, preventing TDP‐43 aggregation could have therapeutic potential for ALS/FTD and associated diseases. It has been shown that aggregation of TDP‐43 can be attenuated by chaperone proteins. Herein, we have identificatied and validated a new small molecule 'JRMS' as potent binder of {IP‐protein} which upregulates its non‐canonical chaperone function to prevent/reverse TDP‐43 aggregation.

**Method:**

We developed high throughput models of TDP‐43 aggregation by expressing the highly aggregation prone C‐terminal fragment TDP‐25 in cells through transient expression, mouse primary cortical neurons through lentiviral expression, and organotypic slices and mouse model through AAV9 expression.

**Result:**

Acute and chronic treatment of JRMS reduced TDP‐25 aggregation in a dose‐dependent manner by ∼75% in cells. This reduction of TDP‐25 aggregates was validated to be dependent on {IP‐protein}, and that JRMS elevates activity of the target {IP‐protein}. Similarly, in mouse primary cortical neurons transduced with TDP‐25 lentivirus, JRMS reduced TDP‐25 aggregates by ∼50% reduction. We screened JRMS on organotypic slices from 10 day‐old mice inoculated with AAV9‐TDP‐25. Over 7‐days, DMSO treated slices showed ∼60% increase, while JRMS treatment showed a ∼20% reduction in number of TDP‐25 aggregates observed prior to treatment. Finally, two weeks treatments of AAV9‐TDP‐25 expressing mice at 6 weeks of age with JRMS exhibited ∼30% reduction in number of aggregates compared to DMSO control.

**Conclusion:**

We have validated JRMS as a preclinical therapeutic candidate for the treatment of TDP‐43 proteinopathy, with additional preliminary evidence for Tauopathies as well. We are currently working on building a Target Product Profile (TPP) with the goal of providing a drug(s) for clinical testing.

